# Urban Dog Parks as Sources of Canine Parasites: Contamination Rates and Pet Owner Behaviours in Lisbon, Portugal

**DOI:** 10.1155/2017/5984086

**Published:** 2017-08-30

**Authors:** Ana Ferreira, Ana Margarida Alho, David Otero, Lídia Gomes, Rolf Nijsse, Paul A. M. Overgaauw, Luís Madeira de Carvalho

**Affiliations:** ^1^CIISA, Faculty of Veterinary Medicine, Universidade de Lisboa, Avenida da Universidade Técnica, 1300-477 Lisboa, Portugal; ^2^Department of Infectious Diseases and Immunology, Faculty of Veterinary Medicine, Utrecht University, Postbus 80165, 3508 TD Utrecht, Netherlands; ^3^Institute for Risk Assessment Sciences, Division of Veterinary Public Health, Utrecht University, Postbus 80163, 3508 TD Utrecht, Netherlands

## Abstract

Dog parks represent a recent trend in western countries, enabling owners to spend quality time with their pets in a controlled environment. Despite their growing popularity, few studies have been performed to date on these parks to investigate dog intestinal parasitic infections and soil contamination. The present study examined 369 faecal and 18 soil samples collected from 3 dog parks in Greater Lisbon, Portugal. Additionally, 102 interviews were performed with dog owners to assess dog-walking behaviours and parasite risk. In total, 33% of the faecal dog samples were infected with at least one parasitic agent: hookworms (16.5%),* Cryptosporidium* spp. (11.9%),* Giardia* spp. (11.4%),* Toxascaris leonina* (1.1%),* Cystoisospora* spp. (1.1%),* Toxocara* spp. (0.5%), and* Sarcocystis* sp. (0.3%). The soil of all the parks was contaminated with hookworm eggs. This is the first study performed in a European urban area to assess canine faecal contamination and parasitic agents in dog parks. Our results highlight the potential of these parks as a source of transmission for canine parasites, including some with zoonotic potential. Public awareness and effective preventive measures should be promoted to minimise the health-risk impact to both animals and humans, under the scope of environmental and public health.

## 1. Introduction

In modern-day society, the human-animal bond has become stronger with pets playing an important role as a source of companionship, emotional support, and recreation. Dogs encourage easier social interactions between people and promote the physical and psychological health of their owners [1, 2]. Despite the positive effects that pets can have on people's lives, this close bond may also compromise human health due to allergic reactions, trauma, and infectious diseases [2]. Dogs have been implicated in the transmission of more than 60 zoonotic infectious diseases [1], some of which are due to canine intestinal parasitic infections and are of serious concern. For instance,* Toxocara* spp. are responsible for visceral larva migrans (VLM), ocular larva migrans (OLM), covert toxocariasis, and neurological and atopic signs [3]. Some hookworms can cause cutaneous larva migrans (CLM) and eosinophilic enteritis [4]. Assemblages A and B in the protozoan genus* Giardia* are considered to have a zoonotic potential [5], and the risk of human infection by* Cryptosporidium* sp. from dogs, though limited, has not been excluded [6]. Until now, children, pregnant women, elderly, and immunocompromised people are all at a higher risk of disease resulting from parasitic zoonoses [7].

Dog parks (i.e., enclosed areas for domesticated dogs to play off-leash and socialise with other canines in a controlled environment) enable owners to spend quality time with their dogs. These areas promote social interactions among dogs and offer a safe setting for regular exercise in a controlled environment under the supervision of their owners [8]. Over the last decades, dog parks became very popular in urban areas and are one of the fastest growing segments of city parks. However, these parks may pose an increased risk for the transmission of parasitic zoonotic agents, via faecal and soil contact, among dogs, humans, and wildlife [8, 9]. Despite this, few studies investigating soil contamination and intestinal parasitic infections in dog parks have been carried out thus far, including one in Colorado, USA [10], and a second in Calgary, Alberta, Canada [8]. No studies have been performed in urban dog parks in Europe to date.

In order to assess faecal environmental contamination and intestinal parasites in frequenting purpose-built dog parks, faecal and soil samples were collected from such parks located in urban areas of Lisbon, Portugal. Interviews were conducted to assess owners' behaviour, veterinary care, and the owner-pet relationship.

## 2. Materials and Methods

### 2.1. Sampling Area

**Figure 1:**
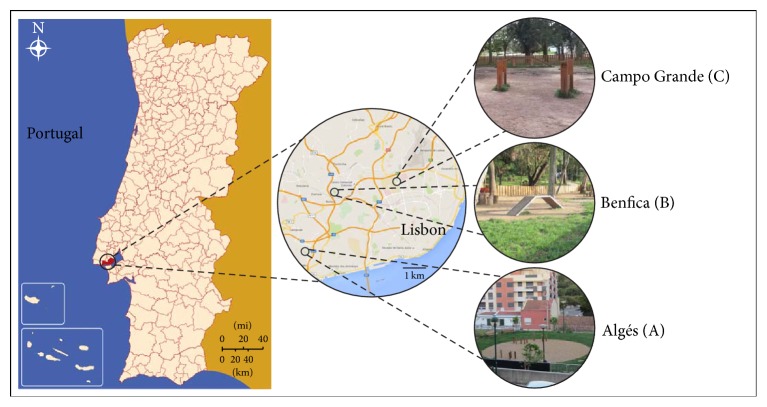
Map highlighting the three dog parks assessed in Greater Lisbon, Portugal.

Three purpose-built dog parks located in Greater Lisbon were chosen, in order to represent this type of facility located in densely populated areas distributed throughout the city: (A) Algés, a 350 km^2^ parish with 48.665 inhabitants and 1.693 licensed dogs; (B) Benfica, a 465 km^2^ parish with 36.821 inhabitants and approximately 400 licensed dogs; and (C) Campo Grande, a 1.120 km^2^ parish with 31.813 inhabitants and about 1.700 licensed dogs (Figure 1). The dog parks were fenced with a double-gated entry and had shades, drainage, water sources, and covered garbage cans. All parks were regularly maintained, including ground cleaning. This study was conducted from October to December 2015. For a better representation of the visiting population, fresh faecal samples randomly distributed throughout the parks were collected every 15 days, at different periods of the day (morning, midday, and afternoon). Once a month over the same period, soil samples were collected from five distinct spots with a gardening spade and subsequently pooled (approximately 250 grams). Samples were obtained from a depth of between 0 and 5 cm of grass (3 samples/park) and gravel (3 samples/park). Although cleaning and disinfection activities are implemented on a regular basis, the authors were unaware of any of them performed on the surveyed parks immediately prior to sampling dates.

Over the same period, 102 owners walking their dogs in the three dog parks were interviewed.

### 2.2. Coprological Analysis

#### 2.2.1. Parasite Egg Isolation and Identification

A Centrifugal Sedimentation Flotation (CSF) technique was used [11]. Briefly, 3–5 g of each faecal sample was homogenised in 55 ml of distilled water and sieved through a tea strainer into a tube. Tubes were centrifuged (3 minutes at 3000 rpm) and the supernatant was discarded. A third of the tube was filled with sucrose solution (specific density: 1.3 g ml^−1^), vortexed, filled again with sucrose solution, and centrifuged (3 minutes at 3000 rpm). Tubes were then filled with sucrose solution until a convex meniscus was formed and a coverslip was then placed immediately on the top. After 25 minutes, the coverslip was placed on a slide for observation using an optical microscope at 100x–400x magnification. Eggs, oocysts, and cysts were identified morphologically according to published guides [12–14]. Names of the parasites of genus* Cystoisospora* followed a recent taxonomic revision [15].

#### 2.2.2. Faecal Smears

A faecal smear, stained by the modified Ziehl-Neelsen technique [16], was performed on each sample. Briefly, a small amount of faeces was spread over a slide to form a thin layer, using a stirring rod. After drying, smears were fixated with methanol for 1 minute, covered with fuchsine for 10 minutes, and washed under running water. They were subsequently washed with 1% hydrochloric alcohol to remove excess fuchsine and washed again with running water. Slides were then covered with 0.4% malachite green for 30 seconds, washed again with running water, and finally left to air-dry. Smears were observed at 1000x magnification for the detection of* Giardia* sp. cysts, as transparent oval bodies with 4 nuclei, and* Cryptosporidium* sp. oocysts, as round oocysts frequently containing typical crescent shaped sporozoites stained with a pink-reddish colour. A minimum of 50 fields were observed per slide. Genotyping of* Giardia* spp. and* Cryptosporidium* spp. isolates was not possible to perform.

### 2.3. Soil Analysis

Soil samples were analysed using a modified Sieving and Centrifugal Sedimentation Flotation (CSF) technique [17, 18]. For each sample, one hundred grams of soil was weighed, mixed with 100 mL of 5% Tween-20 solution, and then homogenised for 10 minutes and allowed to stand overnight. The contents were then sieved (diameters 1.000 mm, 0.500 mm, 0.250 mm, 0.150 mm, 0.063 mm, and 0.020 mm) and washed under running water for 30 minutes. The sediment present in the 0.063 mm and 0.020 mm sieves was resuspended in distilled water and allowed to stand overnight. The sediment was then resuspended with distilled water and the tubes were centrifuged for 3 minutes at 2000 ×g. The supernatant was discarded and the parasite eggs were collected from sediment and identified as mentioned above.

### 2.4. Interviews

Multiple-choice questionnaires were completed as oral face-to-face interviews conducted with 102 dog owners walking their dogs in each one of the three dog parks. Overall, 34 questionnaires were performed per park. Owners were asked several questions intended to assess dog-walking behaviours, including park visitation frequency, animal healthcare, and dog-owner habits.

### 2.5. Statistical Analysis

Statistical analysis was performed using R, version 3.1.3, and the extension R Commander (the R Foundation for Statistical Computing, 2013). Chi-square or Fisher's exact tests were used to compare proportions and a probability *p* value < 0.05 was considered as statistically significant. Exact binomial 95% confidence intervals (CI) were established for proportions.

## 3. Results

### 3.1. Faecal Samples

In total, 369 faecal samples were collected: 125 from Algés (A), 124 from Benfica (B), and 120 from Campo Grande (C). A total of 18 soil samples were also collected, 6 from each park (3 from grass and 3 from gravel surfaces). The overall prevalence of various parasites in the faecal samples from the three different parks is presented in Table 1.

**Figure 2:**
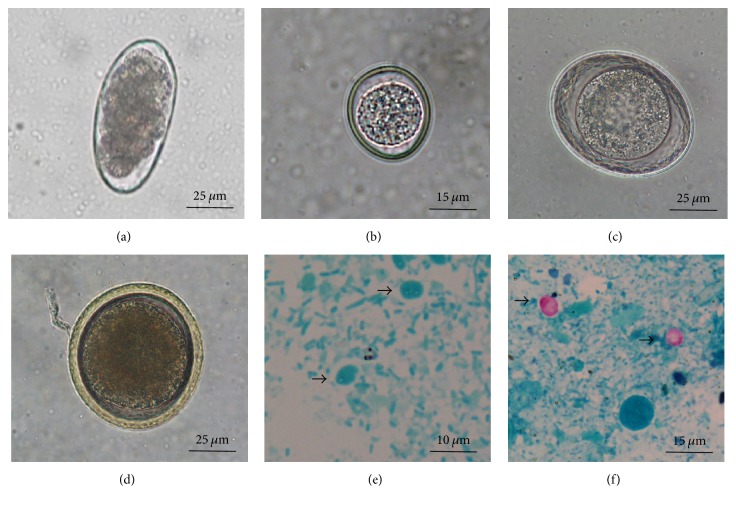
Some of the eggs, cysts, and oocysts detected in fresh faecal samples collected from dog parks using Centrifugal Sedimentation Flotation technique and faecal smears stained by the modified Ziehl-Neelsen technique. (a) Morulated hookworm egg; (b)* Cystoisospora* spp. unsporulated oocyst; (c) nonembryonated* Toxascaris leonina* egg; (d) nonembryonated* Toxocara canis* egg; (e)* Giardia* spp. cysts in faecal smear (arrows); (f)* Cryptosporidium* spp. oocysts in faecal smear (arrows).

Hookworms were the most prevalent group of parasites detected.* Cryptosporidium* spp.,* Giardia* spp., and* Cystoisospora* spp. were also identified in all three parks whereas* Toxocara* spp. were detected in only two parks and* Toxascaris leonina* and* Sarcocystis* sp. were detected in only one. Dog park A showed a greater biodiversity in its parasitic fauna with 6 of the 7 parasite groups diagnosed, whereas dog parks B and C had only 5 different types of parasites (Figure 2). No significant statistical differences were detected between parks (*p* = 0.81).

### 3.2. Soil Samples

In total, 18 soil samples were collected, 6 per each park (3 from grass and 3 from gravel). Five of the 9 samples (55.6%) from grassed areas contained hookworm eggs (Ancylostomatidae) whereas 0/9 samples from gravel areas had hookworm eggs, showing a significant statistical difference between grassed and gravel areas (*p* = 0.03). Overall, 27.8% (5/18) of the soil samples (all from grassed areas) were contaminated with hookworm eggs, in the three assessed parks: 33.3% (CI 6.0–75.9%) from A, 16.7% (CI 0.9–63.5%) from B, and 33.3% (CI 6.0–75.9%) from C. Eggs were only found in grassed areas. Regarding soil samples, no significant statistical differences were detected between the parks (*p* = 1).

### 3.3. Interviews

It was found that 40.2% of the dogs present in the parks live in a home/dwelling with at least one other animal (most with other dogs and cats and a minority with birds, rabbits, or guinea pigs). Regarding daily walking, 82.3% were walked both on the streets and in parks, 16.7% only in parks, and 1.0% in parks and open field. In addition, 41.2% of the respondents mentioned visiting with their dogs more than one of the dog parks located in Lisbon.

Of the total respondents, 50.0% visited the park daily, 29.4% at least once a week, and 20.6% less than 1–3 times a month. Most dog owners walked their dogs off-leash (57.8%), 17.6% on-leash, and 24.5% both (82.3% with off-leash activity, overall). Almost all owners, 94.1%, claimed to collect their dog's faeces.

Regarding animal healthcare, 93.1% of dog owners answered to have taken their dog to a veterinarian consultation in the previous 12 months. Concerning anthelmintic treatments, 89.9% of the owners stated to have internally dewormed their dogs in the previous six months. However, when asked regarding its specific frequency, 14.5% answered three times a year, 41.0% twice a year, 13.3% once a year, and only 27.7% at least four times a year.

The most commonly used anthelmintic drug (72.7% of respondents) was the combination of praziquantel-pyrantel embonate with a third molecule (febantel, oxantel, or fenbendazole).

In 82.4% of the households, the dog was allowed to visit the owners' bedroom, 75.5% were allowed to lick their owners' faces, and 43.1% were allowed to sleep with the owners in their beds.

## 4. Discussion

This is the first study performed to assess canine faecal contamination and parasitic agents in urban dog parks and dogs frequenting such parks in Europe. The three parks had similar rates of contamination, with one-third of dog faecal samples positive for at least one parasite. Hookworms were the parasite group detected with the highest prevalence (16.5%) in all sampled parks. In Europe, there are two main species of hookworms:* Ancylostoma caninum* (the potentially zoonotic helminth responsible for cutaneous larva migrans) and* Uncinaria stenocephala* (nonzoonotic). As faecal culture and larvae examination were not performed, these two species were not differentiated and zoonotic potential could not be assessed. Although faecal samples were fresh, they were directly exposed to sunlight and warm temperatures for some hours until collection. Indeed, according to Anderson (2000) [19], the embryo formation of* A. caninum* eggs easily takes place and greatly varies with environmental temperature, ranging from 6–12 days at 12°C to 10–12 hours at 30°C. Similarly,* U. stenocephala* eggs can hatch within 12 hours at 20°C. Considering the high temperatures registered in Lisbon during the study sampling period, this fact explains why the authors found several embryonated eggs, despite working with fresh collected samples.

Concerning the percentage of hookworm eggs contaminating the herbage (55.6%), it mirrors the results found also by other authors concerning both domestic and wild canids. Hookworm infections had been commonly reported in household, hunting, kennel, and farm canids from north and south of Portugal, being the predominant helminth eggs detected [20, 21]. Although free-living nematodes may also be found when sampling herbage, they were distinguished by the characteristics of the adult stages (e.g., rhabditids) and by their eggs which are smaller and more transparent. Additionally, hookworm samples were found in parks where neither rabbits nor rodents were found, given the regular cleaning measures and rodent control program performed by municipal city services in these areas. In the present study, hookworm eggs were the only parasites found in soil samples, which suggests recent contamination, as these eggs do not generally persist for long periods in the environment [22]. Additionally, only grass samples were positive for parasite ova. The lack of ova in gravel samples is possibly explained by the large size of the gravel grains that do not retain parasitic elements, or by the fact that dogs prefer to defaecate on grass rather than on gravel. Furthermore, grass areas protect more the eggs from direct sun exposure in comparison with sandy areas, where eggs may have been destroyed by desiccation after direct sunlight exposure.

Protozoa were also found in all sampled parks, being more prevalent than nematodes. Indeed, a declining trend in the prevalence of intestinal helminths has been observed in certain countries over the last few decades, possibly explained by owner's increased awareness and consequent application of routine preventive anthelmintic treatments [7]. Such anthelmintics usually do not have label claims that include intestinal protozoa [7].

In the present study, oocysts of* Cryptosporidium* spp. were found in 11.9% of the faecal samples. This prevalence is in between the 4.8% of park-attending dogs from Colorado, United States of America [10], and the 14.7% detected in park-attending dogs from Calgary, Canada [8]. Lower prevalence was detected in other European countries, such as 0% in Belgium [23] and 2.6% in France [24].* Cryptosporidium* infection in dogs is mainly caused by* Cryptosporidium canis* whereas in humans it is mainly due to* Cryptosporidium hominis*. In fact, the risk of humans acquiring the infection from dogs seems to be minimal, mostly limited to immunocompromised individuals, although zoonotic potential has not been conclusively ruled out by the scientific community [6].

*Giardia* spp. were found in 11.4% of the faecal samples, again an intermediate value between the 7.6% in park-attending dogs from Colorado [10] and the 24.7% from Calgary [8], performed with direct immunofluorescence assay, a more sensitive method of detection.* Giardia* spp. trophozoites and cysts were searched using CSF and Ziehl-Neelsen staining of faecal smears. Although the latter technique is not much referred for detection of* Giardia* spp., it was chosen in this study because of its common use for the diagnosis of* Cryptosporidium* spp., allowing the simultaneous detection of both agents. This is an easy and well-suited detection method for general practice [25]. Similar studies conducted in other areas of Portugal using zinc sulphate showed prevalence of 7.4% in asymptomatic dogs and 15.5% in symptomatic dogs from Oporto [26], and 1.3% in household dogs and 61.2% in kennel dogs from Évora [21].

In fact,* Cryptosporidium* spp. and* Giardia* spp. are frequently associated with waterborne outbreaks. In a study conducted in Lisbon to assess the presence of* Cryptosporidium* and* Giardia* in raw and treated water by immunofluorescence (IFA) microscopy and PCR,* Cryptosporidium* spp. oocysts were found in 53.6% of untreated and in 41.5% of treated water samples, whereas* Giardia* spp. cysts were detected in 58.0% of untreated and in 25.6% of treated water samples [27].

Although there is only one species of* Toxocara* in the dog* (Toxocara canis)*, as dogs may also shed eggs of* Toxocara cati* due to coprophagy and as morphological distinction between* T. canis* and* T. cati* eggs is very difficult with light microscopy, the authors only indicated the genus. The prevalence of dogs shedding* Toxocara* eggs in the present study was low, particularly when compared to the results found by Otero et al. [28] who detected 63.2% of soil and 15.8% of faecal samples positive for* Toxocara* spp. in urban public parks and children playground sandpits of Lisbon. A higher prevalence of dogs shedding* Toxocara* eggs was detected in a study performed in Oporto, Portugal, using zinc sulphate, where 5.1% of asymptomatic dogs and 7.8% of gastrointestinal symptomatic dogs admitted to a veterinary hospital tested positive [26]. Nevertheless, the prevalence detected in the present study might be underestimated as several parasites (in particular* Toxocara canis*) affect mainly puppies, which are not taken to public places or spaces of canine socialisation, because they have not yet been fully vaccinated. This might also be the justification for the low prevalence (1.1%) of samples positive for* Cystoisospora* spp., a protozoan that is mostly found in puppies, usually not taken to public places or dog parks. A higher prevalence of* Cystoisospora* spp. was found in Oporto (13.5%) in dogs presenting gastrointestinal signs [26]. Other studies performed in Europe reveal higher prevalence, such as central Italy (7.5%) [29] and Spain (10.2%) [30].

*T. leonina* was also found in 1.1% of the samples, a similar prevalence to the 0.5% detected in dogs with gastrointestinal signs from Oporto [26].

Sporocysts of* Sarcocystis* spp. were found in only one sample (0.3%), a very low prevalence, possibly explained by the indirect life cycle of this parasite. This parasite was also diagnosed in domestic canids in other researches carried out in Northern Portugal and its prevalence rates were also low [31].

Regarding* Trichuris vulpis*, heavy infections tend to be geographically localised or to occur mostly in kennels [32], which might explain the lack of positive results for this parasite in the present study.

The high prevalence of detected helminths generally covered by regular deworming products suggests that few dogs are internally dewormed with the recommended schedule (minimum quarterly) [32] despite the frequent contact with other animals. Indeed, the percentage of dewormed dogs in this study is in agreement with Matos et al. [33], who observed that although the majority of Portuguese pet owners give antiparasitic drugs to their pets, most of them do not follow the manufacturer's recommendations and veterinary advice, deworming at irregular intervals.

According to Smith et al. [8], infection with enteric parasites is positively associated with off-leash activity, park visitation frequency, and visiting more than one park. In the present study, 82.3% had off-leash activity, 50.0% of the dogs visited dog parks daily, and 41.2% frequented other parks. Additionally, approximately 40% of the surveyed dogs shared the house with other animals and less than one-third were dewormed according to the recommended regimen. Although 94.1% of the owners stated that they collect their pet's faeces, 5 to 10 faecal samples were spotted by the authors in each dog park, every sampling date (Ana Ferreira, personal communication). In the study of Matos et al. [33], 63.3% of the Portuguese dog owners affirmed collecting their dogs' faeces in public areas, 95.6% whenever this occurs on a city path or pavement and 82.9% whenever this occurs in city parks. These results match the 94.1% of the owners who stated that they collect their pet's faeces in the present study. Nevertheless, it could be possible that the percentage found in our study may be overestimated, not reflecting owners' real behaviour, as this is a sensitive matter and data was not collected anonymously. Still, this measure should be encouraged, as it is an extremely important and easy way to reduce environmental contamination to safeguard public and animal health.

Despite the prevalence of the various parasites detected in these dog parks, the present results should be interpreted with caution, as some limitations should be pointed out. Multiple sampling of the same animal(s) cannot be excluded because the source of each faecal sample was not known. In addition, it was not possible to pair survey findings to faecal samples on an individual basis, which hampers the capacity to assess the risk factors of this population. Larvae examination after faecal culture, assessment of* Toxocara* spp. egg infection ability, and genotyping would have been particularly relevant to determine the hookworm species, the zoonotic potential of* Toxocara* eggs, and the genotypes of* Giardia* spp. and* Cryptosporidium* spp. isolates and, consequently, the potential zoonotic impact of these parasites. The sample size was small regarding the number of samples and geographic distribution, hindering an inference to the whole area of Lisbon. However, one-third of faecal samples with at least one parasite, using the above-mentioned techniques, must be considered a relevant finding for a supposedly well-controlled dog population regarding canine gastrointestinal parasitic diseases, according to the owner's answers. For this reason, further studies are needed involving larger samples and other geographic areas in Portugal, to better understand the potential of dog parks as a transmission source of parasitic diseases for animals and humans.

## 5. Conclusions

In conclusion, soil contamination with the potentially zoonotic hookworm eggs was present in all the parks assessed. In addition, one-third of dog faecal samples contained detectable parasites, including two nematodes with potential zoonotic impact (hookworms and* Toxocara* spp.) and two potentially zoonotic genera of protozoa (*Cryptosporidium* spp. and* Giardia* spp.). Further studies are needed to assess if such risks are present in other dog parks, located in other cities in our country, and all over Europe. Despite being considered for many owners as a destination of excellence for their dogs, these results highlight the potential of dog parks as a source of transmission of several parasitic diseases, especially when considering the high level of human and canine movement in such confined areas. This is particularly likely when appropriate cleaning measures and effective deworming practices are lacking. Additionally, the close physical contact and some behavioural practices reported by several owners not only show a lack of knowledge regarding animal and public health issues but also can pose an increased risk for the transmission of zoonotic diseases. Public awareness and effective preventive measures should be promoted, to minimise the health-risk impact to both animals and humans, under the scope of environmental and public health.

## Figures and Tables

**Table 1:** Prevalence of the parasites detected in faecal samples collected in three dog parks (A, B, and C) of Greater Lisbon, Portugal.

	A (*n* = 125) (95% CI)	B (*n* = 124) (95% CI)	C (*n* = 120) (95% CI)	Total (*n* = 369) (95% CI)
Hookworms	14.4% (9.0–22.1)	18.5% (12.4–26.7)	16.7% (10.7–24.8)	16.5% (13.0–20.8)
*Cryptosporidium *spp.	12.0% (7.1–19.3)	15.3% (9.7–23.2)	8.3% (4.3–15.2)	11.9% (8.9–15.8)
*Giardia* spp.	16.0% (10.3–23.9)	6.5% (3.0–12.7)	11.7% (6.8–19.1)	11.4% (8.4–15.2)
*Cystoisospora* spp.	0.8% (0.0–5.0)	1.6% (0.3–6.3)	0.8% (0.0–5.2)	1.1% (0.4–2.9)
*Toxascaris leonina*	0	0	3.3% (1.1–8.8)	1.1% (0.4–2.9)
*Toxocara* spp.	0.8% (0.0–5.0)	0.8% (0.0–5.1)	0	0.5% (0.1–2.2)
*Sarcocystis* sp.	0.8% (0.0–5.0)	0	0	0.3% (0.0–1.7)

Total of positive samples	35.2% (27.0–44.3)	31.5% (23.6–40.5)	32.5% (24.4–41.7)	33.1% (28.3–38.2)
